# The Current State and Diagnostic Accuracy of Digital Mental Health Assessment Tools for Psychiatric Disorders: Protocol for a Systematic Review and Meta-analysis

**DOI:** 10.2196/25382

**Published:** 2021-01-08

**Authors:** Nayra A Martin-Key, Thea S Schei, Eleanor J Barker, Benedetta Spadaro, Erin Funnell, Jiri Benacek, Jakub Tomasik, Sabine Bahn

**Affiliations:** 1 Department of Chemical Engineering and Biotechnology Cambridge Centre for Neuropsychiatric Research University of Cambridge Cambridge United Kingdom; 2 Psyomics Ltd Cambridge United Kingdom; 3 University of Cambridge Medical Library University of Cambridge Cambridge United Kingdom

**Keywords:** diagnostic accuracy, digital mental health, digital questionnaire, meta-analysis, psychiatry, systematic review

## Abstract

**Background:**

Despite the rapidly growing number of digital assessment tools for screening and diagnosing mental health disorders, little is known about their diagnostic accuracy.

**Objective:**

The purpose of this systematic review and meta-analysis is to establish the diagnostic accuracy of question- and answer-based digital assessment tools for diagnosing a range of highly prevalent psychiatric conditions in the adult population.

**Methods:**

The Preferred Reporting Items for Systematic Review and Meta-Analysis Protocols (PRISMA-P) will be used. The focus of the systematic review is guided by the population, intervention, comparator, and outcome framework (PICO). We will conduct a comprehensive systematic literature search of MEDLINE, PsychINFO, Embase, Web of Science Core Collection, Cochrane Library, Applied Social Sciences Index and Abstracts (ASSIA), and Cumulative Index to Nursing and Allied Health Literature (CINAHL) for appropriate articles published from January 1, 2005. Two authors will independently screen the titles and abstracts of identified references and select studies according to the eligibility criteria. Any inconsistencies will be discussed and resolved. The two authors will then extract data into a standardized form. Risk of bias will be assessed using the Quality Assessment of Diagnostic Accuracy Studies-2 (QUADAS-2) tool, and a descriptive analysis and meta-analysis will summarize the diagnostic accuracy of the identified digital assessment tools.

**Results:**

The systematic review and meta-analysis commenced in November 2020, with findings expected by May 2021.

**Conclusions:**

This systematic review and meta-analysis will summarize the diagnostic accuracy of question- and answer-based digital assessment tools. It will identify implications for clinical practice, areas for improvement, and directions for future research.

**Trial Registration:**

PROSPERO International Prospective Register of Systematic Reviews CRD42020214724; https://www.crd.york.ac.uk/prospero/display_record.php?ID=CRD42020214724.

**International Registered Report Identifier (IRRID):**

DERR1-10.2196/25382

## Introduction

Mental health disorders represent the leading cause of disability worldwide, with over a third of the world’s population being affected by a mental health condition in their lifetime [1]. Despite the well-documented economic and global burdens of mental disorders and the wide range of existing evidence-based treatments, mental health conditions remain largely underdiagnosed or misdiagnosed and undertreated [2,3], even in high-income countries [4,5]. Critically, the challenges associated with identifying and treating mental health disorders are multifaceted and present with a combination of patient, provider, and system-level barriers. With increasing pressure on mental health care budgets and the overwhelming growing burden of mental health disorders globally [6], prevention strategies and improvements in early identification are essential.

In this regard, digital technologies may offer an innovative and cost-effective way to improve and develop mental health care detection and diagnosis. In fact, digital assessment tools have the potential to support health care professionals in the recognition of mental health symptoms and patient-specific treatment needs. Furthermore, the use of digital technologies could help alleviate the load on the health care system by reducing the number of in-person appointments and providing patients with subclinical or mild mental health symptoms with self-help strategies and psychoeducation [7]. Digital solutions for psychiatry also have the potential to lessen some of the barriers associated with disclosing mental health difficulties in person, such as shyness and discomfort, as well as issues related to stigma and discrimination. Furthermore, such technologies can overcome geographical barriers to health seeking and treatment and can facilitate the engagement of conventionally hard-to-reach groups.

Studies have revealed the acceptability and efficacy of digital platforms for improving the reach, quality, and impact of mental health care [8], and patients have been found to value the ease of access and empowerment that can be obtained via the use of a digital platform [9]. Importantly, research has demonstrated that patients have a strong interest in using digital technologies to help monitor their mental health [10,11] and are more likely to report severe symptoms on technology platforms than in a face-to-face meeting with a health care professional [11]. Despite the benefits and potential identified by global and national organizations, such as the World Health Organization (WHO), the National Health Service (NHS), and the US Department of Health and Human Services [12], the implementation of these technologies in public and private mental health care services has been slow.

This may be, in part, due to resistance from medical professionals and public policy makers who may be unaware of how to best integrate the technologies into standard care practices. An area that has received less resistance is that of the digitalization of psychiatric questionnaires, with studies demonstrating comparable interformat reliability relative to traditional pen-and-paper questionnaires [13,14]. While the digitalization of existing psychiatric questionnaires is ongoing, the development of more sophisticated question- and answer-based digital solutions for psychiatry, including the use of audio and video [15,16] and personalized user journeys via dynamic question selection [17], represents a promising ground for further innovation.

Critically, while digital psychiatric questionnaires and other technology-based tools are likely to play an important role in the future of mental health care, little attention and effort have been put into establishing their diagnostic accuracy. To this end, there is a need for a comprehensive appraisal of the current state and diagnostic accuracy of digital solutions for screening and diagnosing mental health conditions. We aim to conduct a systematic review and meta-analysis of available question- and answer-based digital mental health tools for a range of psychiatric conditions in the adult population and to evaluate their diagnostic accuracy. Implications for clinical practice, policy making, development, and innovation will be provided. Additionally, potential routes for improving and facilitating blended care (ie, the combination of traditional and digital services) will be investigated, and directions for future research will be identified.

## Methods

### Overview

This review has been registered with the International Prospective Register of Systematic Reviews (PROSPERO; CRD42020214724). The protocol was developed to comply with the recommendations of the Preferred Reporting Items for Systematic Review and Meta-Analysis Protocols (PRISMA-P) [18]. In line with the PRISMA checklist recommendations, the focus of the systematic review is guided by the population, intervention, comparator, and outcome framework (PICO). This review will involve literature search, article selection, data extraction, quality appraisal, data analysis, meta-analysis, and data synthesis. Protocol amendments will be tracked and reported in the final publication.

### Eligibility Criteria

We aim to conduct a systematic review and meta-analysis of available question- and answer-based digital mental health assessment tools for a range of psychiatric conditions in the adult population and to evaluate their diagnostic accuracy. To do this, the below-mentioned PICO framework will be used.

#### Population

The scope of this research includes a comprehensive range of highly prevalent psychiatric conditions that are typically diagnosed and treated in primary and/or secondary care settings (see Multimedia Appendix 1 for an overview of the lifetime prevalence and patient impact of the concerned conditions). The population will include adults who have been assessed for the presence of any of the following mental health conditions: mood/affective disorders (eg, bipolar disorder and depressive disorders/dysthymia), anxiety disorders (eg, generalized anxiety disorder, social anxiety disorder/social phobia, and panic disorder), trauma and stress-related disorders (eg, posttraumatic stress disorder, acute stress disorder, and adjustment disorder), neurodevelopmental disorders (eg, attention-deficit/hyperactivity disorder and autism spectrum disorders), eating disorders (eg, anorexia nervosa and bulimia nervosa), personality disorders (eg, borderline personality disorder and emotionally unstable personality disorder), substance-related disorders (eg, alcohol use disorder and substance use disorder), obsessive-compulsive disorder, insomnia, and schizophrenia. In consultation with a psychiatrist (SB) and given their relevance for the assessment of the above-listed psychiatric conditions, the following transdiagnostic symptom domains will also be included: self-harm, suicidality, and psychosis.

Studies comprising age ranges, where the mean age falls within 18 to 65 years, will be included. The review will focus on both clinical and community-based samples of any gender, severity of mental health concern, ethnicity, and geographical location.

#### Intervention

Interventions of interest include question- and answer-based digital diagnostic tools completed by an individual that a health care professional might use to reach a mental health diagnosis. This can comprise pen-and-paper psychiatric questionnaires that have been digitalized and digital assessment tools that are intended to aid in clinical decision-making, including script-based automated conversational agents (ie, chatbots). The format of delivery can include computerized or web-based interventions delivered either offline or online via a computer, tablet, or smartphone.

#### Comparator

No specific comparator is required for studies to be included in this systematic review and meta-analysis.

#### Outcomes

The primary objectives are to identify the types of question- and answer-based digital assessment tools used in mental health care and to assess their diagnostic accuracy (eg, sensitivity and specificity).

#### Study Design

We will consider any study design for the assessment.

### Search Strategy

We will search the following databases: MEDLINE, PsychINFO, Embase, Web of Science Core Collection, Cochrane Library, Applied Social Sciences Index and Abstracts (ASSIA), and Cumulative Index to Nursing and Allied Health Literature (CINAHL). Other potentially eligible trials or publications will be identified by hand searching the reference lists of retrieved publications, systematic reviews, and meta-analyses. Grey literature (eg, unpublished theses, reports, and conference presentations) will also be identified by hand. Keywords and subject headings related to digital technologies, assessment tools, and diagnostic accuracy outcomes were identified in a preliminary scan of the literature and chosen in consultation with a medical librarian (EB). Key terms for the most common mental health conditions were taken from the Diagnostic and Statistical Manual of Mental Disorders (DSM)-5 and International Classification of Diseases (ICD)-11 (or DSM-IV and ICD-10 for older publications) diagnostic manuals and chosen in consultation with a psychiatrist (SB). In addition to these, notable symptom domains, such as self-harm, suicidality, and psychosis, were included in the search terms on the basis of their relevance in psychiatric assessments. The search terms that will be included in this review are grouped into four themes and are presented in Table 1, with search strategies presented in Multimedia Appendix 2. For simplicity, while we will not specifically search for conditions, such as generalized anxiety disorder, separation anxiety disorder, and histrionic personality disorder, these will be captured by our broader search strategy terms (ie, “anxiety disorder” and “personality disorder”). If additional relevant keywords or subject headings are identified during any of the electronic searches, we will modify the electronic search strategies to incorporate these terms and document the changes.

**Table 1:** Search terms.

Category	Keywords/subject headings (in the title or abstract)
Digital technology	“Application” OR “chatbot” OR “computer” OR “conversational agent” OR “device” OR “digital” OR “e-health” OR “e-mental health” OR “electronic” OR “internet” OR “mHealth” OR “m-health” OR “mobile” OR “online” OR “PC” OR “phone” OR “smart” OR “tablet” OR “telehealth” OR “telemedicine” OR “text messaging” OR “web” OR “algorithm” OR “software”
Assessment tool	“Assessment” OR “diagnostic” OR “mood diary” OR “PHQ” OR “PHQ-9” OR “GAD” OR “GAD-7” OR “questionnaire” OR “screening” OR “tool” OR “test” OR “The Computerized Adaptive Test for Mental Health” OR “CAT-MH” OR “e-PASS” OR “ WSQ” OR “TAPS” OR “Nview” OR “ada” OR “doctorlink” OR “clinicom”
Mental health	“Depression” OR “major depressive disorder” OR “MDD” OR “dysthymia” OR “bipolar” OR “anxiety disorder” OR “generalised anxiety disorder” OR “generalized anxiety disorder” OR “GAD” OR “panic disorder” OR “social anxiety disorder” OR “social phobia” OR “attention-deficit/hyperactivity disorder” OR “attention deficit hyperactivity disorder” OR “ADHD” OR “autism spectrum disorders” OR “ASD” OR “insomnia” OR “eating disorders” OR “anorexia nervosa” OR “bulimia nervosa” OR “obsessive compulsive disorder” OR “OCD” OR “schizophrenia” OR “psychosis” OR “alcohol abuse” OR “alcohol addiction” OR “substance abuse” OR “substance addiction” OR “drug abuse” OR “drug addiction” OR “post-traumatic stress disorder” OR “PTSD” OR “acute stress disorder” OR “adjustment disorder” OR “personality disorder” OR “borderline personality disorder” OR “BPD” OR “emotionally unstable personality disorder” OR “EUPD” OR “self harm” OR “self-harm” OR “suicidality”
Diagnostic accuracy	“Accuracy” OR “sensitivity” OR “specificity” OR “receiver operating characteristic” OR “ROC” OR “area under the curve” OR “AUC” OR “AUROC” OR “positive predictive value” OR “PPV” OR “negative predictive value” OR “NPV” OR “precision” OR “recall” OR “true positive rate” OR “TPR” OR “true negative rate” OR “TNR” OR “agreement rate” OR “validity”

### Inclusion Criteria

Owing to the recent developments in the digitalization of existing psychiatric questionnaires and the rapid growth in digital assessment tools for the screening and diagnosis of mental health conditions, only studies published in the last 15 years (from January 2005) will be included. Studies that evaluate at least one question- and answer-based digital assessment tool to screen or diagnose one or more mental health conditions covered by this review will be included. Any gender, severity of mental health concern, ethnicity, and geographical location will be included. Any study design will be included.

### Exclusion Criteria

Studies of digital assessment tools that are not exclusively question and answer based, such as blood tests, imaging techniques, monitoring tools, genome analysis, accelerometer devices, and wearables, will also be excluded. Specific subgroups, such as pregnant women, refugees/asylum seekers, prisoners, and those in acute crisis/admitted to emergency services will be excluded. Studies on tools used to identify mental health disorders in physical illnesses (eg, cancer) will also be excluded. We will also exclude studies on somatoform disorders and specific phobias as these are less frequently diagnosed in primary care and rarely present in secondary care. In addition, studies on tools used to identify neuropsychiatric disorders (eg, dementias) or any disorders that are due to clinically confirmed temporary or permanent dysfunction of the brain are outside the scope of the current review. Studies on digital assessment tools used to predict the future risk of developing a mental health disorder will also be excluded.

### Screening and Article Selection

**Figure 1:**
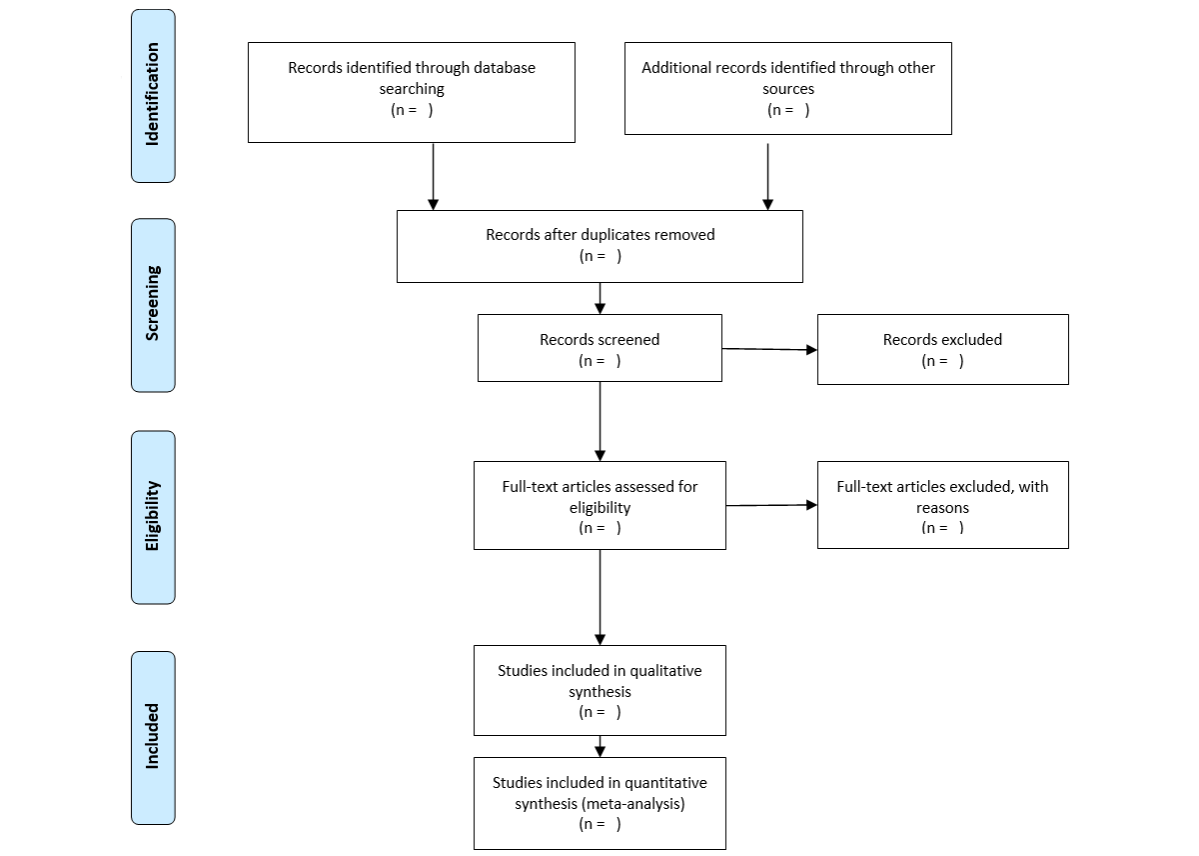
PRISMA flow-chart template of the search and selection strategy.

All articles identified from the database searches will be stored in the systematic review software Rayyan, which will be used to eliminate any duplicates. Two independent reviewers will screen the titles and abstracts of all the studies. To decide whether an article should be examined further, independent reviewers will assess their eligibility against the inclusion criteria. Publications will be labelled as “exclude,” “include,” or “maybe.” For an article to be included, both reviewers must label it as “include.” An article will be excluded if both reviewers label it as “exclude.” Articles labelled as “maybe” or any disagreements will be discussed until a consensus is reached. All exclusions will be documented. The screening process will be piloted and tested by the reviewers on a subset of 100 studies, after which the review will continue. The full text of the “included” articles will then be examined by the two independent reviewers in order to determine final eligibility, with any disagreements being resolved by a third reviewer. All reasons for full-text exclusions will be recorded. A PRISMA flow diagram will be used to record the details of the screening and selection process so that the study can be reproduced (Figure 1).

### Data Extraction

Two independent reviewers will examine the full text of all the papers included in the final selection to extract the predetermined outcomes. Outcomes will be extracted into a predetermined standardized electronic data collation form, and they will include (1) publication details: author(s) and date; (2) study design and methodology: sample size(s), sample characteristics (mean age, proportions of males and females, ethnicity, and geographical location), recruitment and sampling procedures, main psychiatric diagnosis, and how psychiatric diagnosis was established/confirmed; (3) index test (ie, the digital assessment tool) and reference standard (ie, assessment by a psychiatrist and standardized structured and semistructured diagnostic interviews based on the DSM-5 and ICD-11 criteria, or DSM-IV and ICD-10 for older publications, such as the Composite International Diagnostic Interview [19] and the Structured Clinical Interview for DSM-5 Disorders [20]); and (4) outcomes of interest: measure of diagnostic accuracy.

Disagreements will be resolved by discussion, and if consensus cannot be reached, a third reviewer will be consulted.

### Quality Appraisal: Risk of Bias and Applicability

Following the final selection of studies, two independent reviewers will assess risk of bias and applicability of all included studies using the Revised Tool for the Quality Assessment of Diagnostic Accuracy Studies (QUADAS-2 [21]). The checklist consists of the following four key domains: patient selection, index test, reference standard, and flow of participants through the study and timing of the index tests and reference standard. Each of these domains has a subdomain for risk of bias, while the first three have a subdomain for concerns regarding applicability. The subdomains about risk of bias include signaling questions to guide the overall judgement about whether a study is likely to be biased or not. Studies that are judged as “low” on all domains relating to bias or applicability are classed as having “low risk of bias” or “low concern regarding applicability.” On the other hand, studies judged as “high” or “unclear” in one or more domains may be deemed as “at risk of bias” or as having “concerns regarding applicability.”

In the event of a disagreement, the reviewers will discuss before consulting a third reviewer. A table will be created summarizing the risk of bias and applicability of all included studies.

### Data Analysis and Synthesis

The data analytic strategy was developed in consultation with a statistician. We will conduct a descriptive analysis to summarize the extracted data, with studies grouped by target mental health condition (eg, bipolar disorder).

Where possible and in line with the recommendations in the Cochrane Handbook for Systematic Reviews of Diagnostic Test Accuracy [22], we will construct bivariate random-effects meta-analyses to determine the meta-analyzed sensitivity and specificity of each digital assessment tool and all digital assessment tools collectively per target mental health condition. Summary receiver operating characteristic (sROC) curves with accompanying 95% CIs for each digital assessment tool and for all digital assessment tools collectively per condition will be calculated using hierarchical sROC curve meta-analysis methods.

Between-study variance as a result of heterogeneity for each digital assessment tool and all digital assessment tools collectively per target mental health condition will be assessed using Higgins *I^2^* statistic (0%-25%, might not be important; 25%-50%, might represent low heterogeneity; 50%-75%, might represent moderate heterogeneity; 75%-100%, high heterogeneity [23]). To explore the potential sources of heterogeneity, meta-regression analyses using potential predictive covariates will be conducted where possible. In order to explore potential sources of heterogeneity, QUADAS-related factors, such as participant selection, will be used as predictive covariates in the meta-regression analyses. Further, if sufficient data are available, the effects of the following modifiers will be assessed: (1) reference standard (assessment by a psychiatrist, and standardized structured and semistructured diagnostic interviews based on the DSM-5 and ICD-11 criteria, or DSM-IV and ICD-10 for older publications, are considered the gold standard, but these can vary considerably; thus, separate analyses per reference standard will be conducted); (2) population (inpatient or noninpatient); (3) national context (Western or non-Western); (4) gender (male or female); and (5) mode of delivery (smartphone, tablet, or computer).

Importantly, in the event of overlapping populations across studies, subgroup analyses (excluding the smaller studies with shared populations) will be conducted in order to quantify the impact of these on the overall results. Finally, publication bias will be explored by employing the Begg test [24] and Egger test [25] for each digital assessment tool and all digital assessment tools collectively per target mental health condition. Analyses will be conducted in R (R Foundation for Statistical Computing) in consultation with a statistician. Any amendments to the data analytic strategy will be tracked and reported in the final publication.

## Results

The systematic review and meta-analysis commenced in November 2020. Findings are expected by May 2021. This work has been funded by Stanley Medical Research Institute (SMRI; grant number: 07R-1888) and Psyomics Ltd.

## Discussion

A comprehensive systematic review of the literature and meta-analysis will provide a better understanding of the current state of digital assessment tools for mental health and their diagnostic accuracy. Based on the data, we will identify implications for clinical practice, policy making, development, and innovation. Additionally, potential routes for improving and facilitating blended care (ie, the combination of traditional and digital services) will be investigated, and directions for future research will be identified.

## Appendix

Multimedia Appendix 1 Prevalence and patient impact of the concerned psychiatric conditions.

Multimedia Appendix 2 Search strategies.

## Abbreviations

DSM: Diagnostic and Statistical Manual of Mental Disorders
ICD: International Classification of Diseases
PICO: population, intervention, comparator, and outcome
PRISMA-P: Preferred Reporting Items for Systematic Reviews and Meta-Analyses Protocol
QUADAS-2: Quality Assessment of Diagnostic Accuracy Studies-2
sROC: summary receiver operating characteristic
