# Supplementary material for: The Current State and Diagnostic Accuracy of Digital Mental Health Assessment Tools for Psychiatric Disorders: Protocol for a Systematic Review and Meta-analysis
Source: JMIR Res Protoc. 2021 Jan 8;10(1):e25382. doi: 10.2196/25382 (PMC7822724; doi:10.2196/25382)
Supplement: Multimedia Appendix 1 [file resprot_v10i1e25382_app1.docx]

**Appendix 1**

**The Current State and Diagnostic Accuracy of Digital Mental Health Assessment Tools for Psychiatric Disorders: Protocol for a Systematic Review and Meta-Analysis**

**Table S1.** Prevalence and patient impact of the psychiatric conditions concerned

| **Condition** | **Life-time Prevalence** | **Impact on patient** | **Reference** |
| --- | --- | --- | --- |
| Bipolar disorder | 1.3-3.7% | Severe condition. One of the leading causes of disability among young people. Leads to cognitive and functional impairment. Raised mortality, particularly death by suicide. | [1] |
| Major depressive disorder | 2-21% | Severe forms limit psychosocial functioning and diminish quality of life. Raised mortality, particularly death by suicide. | [2] |
| Obsessive-compulsive disorder | 2-3% | Severe and disabling. | [3] |
| Social anxiety disorder | 4% | Disabling condition associated with reduced quality of life. Risk factor for subsequent depressive illness and substance abuse | [4] |
| Separation anxiety disorder | 4.8% | Highly comorbid with other mental disorders and is associated with substantial impairment in role functioning that persists even after controlling for comorbidity. | [5] |
| Generalized anxiety disorder | 4-7% | Disorder causes seriously impaired social and occupational functioning, comorbidity with other disorders, and increased risk for suicide. | [6] |
| Panic disorder | 5% | Disabling condition. Associated with substantial functional morbidity and reduced quality of life. | [7] |
| Autism spectrum disorder | 1% | Associated with mpairments in social communication, repetitive behaviours and restricted interests. | [8] |
| Attention-deficit/hyperactivity disorder | 2.8% | Ongoing pattern of inattention and/or hyperactivity-impulsivity that interferes with functioning or development. | [9] |
| Insomnia | 33% | Reduced physical and mental health.  Can lead to long-term sedative use and addiction. | [10] |
| Schizophrenia | 0.28-0.48% | Psychotic symptoms such as hallucinations, delusions, and thought disorder (unusual ways of thinking), as well as reduced expression of emotions, reduced motivation to accomplish goals, difficulty in social relationships, motor impairment, and cognitive impairment. | [11,12] |
| Eating disorders | 2.2-8.4% | Severe and disabling condition. One of the leading causes of early morbidity among adolescents and young adults. | [13] |
| Emotionally unstable personality disorder | 0.5-5.9% | High comorbidity with other psychiatric disorders. Associated with functional impairment, especially unstable social connections. | [14] |
| Alcohol abuse | 5.4-13.2% | High rate of mortality and social functioning problems. Individuals with alcohol use disorder report higher levels of disability. | [15,16] |
| Substance abuse | 7.9% | Substance abuse is associated with poorer outcomes in comorbid disorders. | [15,17] |
| Post-traumatic stress disorder | 3.9% | Can lead to substance dependence in order to alleviate symptoms of PTSD. Increased risk of developing depression in individuals with PTSD. | [18] |
| Acute stress disorder | 5-20% (following trauma) | Acute stress disorder may be a risk factor for later trauma related disorders, substance use and depression. Can lead to short-term and long-term physical health problems. | [19] |
| Adjustment disorder | 0.9% | Severe mental disorder. Risk of suicide. Risk of substance use. | [20] |

**References**

1. Grande I, Berk M, Birmaher B, Vieta E. Bipolar disorder [Internet]. Lancet. Lancet Publishing Group; 2016 [cited 2020 Nov 17]. p. 1561–1572. PMID:26388529

2. Gutiérrez-Rojas L, Porras-Segovia A, Dunne H, Andrade-González N, Cervilla JA. Prevalence and correlates of major depressive disorder: a systematic review. Brazilian J Psychiatry [Internet] FapUNIFESP (SciELO); 2020 Aug 3 [cited 2020 Nov 17];(AHEAD). [doi: 10.1590/1516-4446-2020-0650]

3. Abramowitz JS, Taylor S, McKay D. Obsessive-compulsive disorder [Internet]. Lancet. Lancet; 2009 [cited 2020 Nov 17]. p. 491–499. PMID:19665647

4. Stein DJ, Lim CCW, Roest AM, de Jonge P, Aguilar-Gaxiola S, Al-Hamzawi A, Alonso J, Benjet C, Bromet EJ, Bruffaerts R, de Girolamo G, Florescu S, Gureje O, Haro JM, Harris MG, He Y, Hinkov H, Horiguchi I, Hu C, Karam A, Karam EG, Lee S, Lepine JP, Navarro-Mateu F, Pennell BE, Piazza M, Posada-Villa J, ten Have M, Torres Y, Viana MC, Wojtyniak B, Xavier M, Kessler RC, Scott KM, Al-Kaisy MS, Andrade LH, Borges G, Bunting B, de Almeida JMC, Cardoso G, Cia AH, Chatterji S, Degenhardt L, Demyttenaere K, Fayyad J, Hu C yi, Huang Y, Kawakami N, Kiejna A, Kovess-Masfety V, Levinson D, McGrath J, Medina-Mora ME, Moskalewicz J, Pennell BE, Slade T, Stagnaro JC, Taib N, Whiteford H, Williams DR. The cross-national epidemiology of social anxiety disorder: Data from the World Mental Health Survey Initiative. BMC Med [Internet] BioMed Central Ltd.; 2017 Jul 31 [cited 2020 Nov 17];15(1):35. PMID:28756776

5. Silove D, Alonso J, Bromet E, Gruber M, Sampson N, Scott K, Andrade L, Benjet C, De Almeida JMC, De Girolamo G, De Jonge P, Demyttenaere K, Fiestas F, Florescu S, Gureje O, He Y, Karam E, Lepine JP, Murphy S, Villa-Posada J, Zarkov Z, Kessler RC. Pediatric-onset and adult-onset separation anxiety disorder across countries in the world mental health survey. Am J Psychiatry [Internet] American Psychiatric Association; 2015 Jul 1 [cited 2020 Nov 17];172(7):647–656. PMID:26046337

6. Hoge EA, Ivkovic A, Fricchione GL. Generalized anxiety disorder: Diagnosis and treatment [Internet]. BMJ. BMJ; 2012 [cited 2020 Nov 17]. PMID:23187094

7. Roy-Byrne PP, Craske MG, Stein MB. Panic disorder [Internet]. Lancet. Lancet; 2006 [cited 2020 Nov 17]. p. 1023–1032. PMID:16980119

8. Anagnostou E, Zwaigenbaum L, Szatmari P, Fombonne E, Fernandez BA, Woodbury-Smith M, Brian J, Bryson S, Smith IM, Drmic I, Buchanan JA, Roberts W, Scherer SW. Autism spectrum disorder: Advances in evidence-based practice [Internet]. CMAJ. Canadian Medical Association; 2014 [cited 2020 Nov 17]. p. 509–519. PMID:24418986

9. Fayyad J, Sampson NA, Hwang I, Adamowski T, Aguilar-Gaxiola S, Al-Hamzawi A, Andrade LHSG, Borges G, de Girolamo G, Florescu S, Gureje O, Haro JM, Hu C, Karam EG, Lee S, Navarro-Mateu F, O’Neill S, Pennell BE, Piazza M, Posada-Villa J, ten Have M, Torres Y, Xavier M, Zaslavsky AM, Kessler RC, Al-Kaisy M, Subaie A Al, Alonso J, Altwaijri Y, Atwoli L, Auerbach RP, Axinn WG, Benjet C, Bossarte RM, Bromet EJ, Bruffaerts R, Bunting B, Caffo E, de Almeida JMC, Cardoso G, Cia AH, Chardoul S, Chatterji S, Filho AC, Cuijpers P, Degenhardt L, de Graaf R, de Jonge P, Demyttenaere K, Ebert DD, Evans-Lacko S, Fiestas F, Forresi B, Galea S, Germine L, Gilman SE, Ghimire DJ, Glantz MD, He Y, Hinkov H, Huang Y, Karam AN, Kawakami N, Kessler RC, Kiejna A, Koenen KC, Kovess-Masfety V, Lago L, Lara C, Lepine JP, Levav I, Levinson D, Liu Z, Martins SS, Matschinger H, McGrath JJ, McLaughlin KA, Medina-Mora ME, Mneimneh Z, Moskalewicz J, Murphy SD, Nock MK, Oakley-Browne M, Hans Ormel J, Pinder-Amaker S, Piotrowski P, Ruscio AM, Scott KM, Shahly V, Silove D, Slade T, Smoller JW, Stagnaro JC, Stein DJ, Street AE, Tachimori H, Taib N, Have M ten, Thornicroft G, Viana MC, Vilagut G, Wells E, Williams DR, Williams MA, Wojtyniak B. The descriptive epidemiology of DSM-IV Adult ADHD in the World Health Organization World Mental Health Surveys. ADHD Atten Deficit Hyperact Disord [Internet] Springer-Verlag Wien; 2017 Mar 1 [cited 2020 Nov 20];9(1):47–65. PMID:27866355

10. Falloon K, Arroll B, Elley CR, Fernando A. The assessment and management of insomnia in primary care [Internet]. BMJ. BMJ; 2011 [cited 2020 Nov 17]. PMID:21622505

11. Charlson FJ, Ferrari AJ, Santomauro DF, Diminic S, Stockings E, Scott JG, McGrath JJ, Whiteford HA. Global epidemiology and burden of schizophrenia: Findings from the global burden of disease study 2016. Schizophr Bull [Internet] Oxford University Press; 2018 [cited 2020 Nov 17];44(6):1195–1203. PMID:29762765

12. Simeone JC, Ward AJ, Rotella P, Collins J, Windisch R. An evaluation of variation in published estimates of schizophrenia prevalence from 1990-2013: A systematic literature review. BMC Psychiatry [Internet] BioMed Central Ltd.; 2015 Aug 12 [cited 2020 Nov 20];15(1). PMID:26263900

13. Galmiche M, Déchelotte P, Lambert G, Tavolacci MP. Prevalence of eating disorders over the 2000-2018 period: A systematic literature review [Internet]. Am J Clin Nutr. Oxford University Press; 2019 [cited 2020 Nov 20]. p. 1402–1413. PMID:31051507

14. ten Have M, Verheul R, Kaasenbrood A, van Dorsselaer S, Tuithof M, Kleinjan M, de Graaf R. Prevalence rates of borderline personality disorder symptoms: A study based on the Netherlands Mental Health Survey and Incidence Study-2. BMC Psychiatry [Internet] BioMed Central Ltd.; 2016 Jul 19 [cited 2020 Nov 20];16(1). PMID:27435813

15. Kessler RC, Berglund P, Demler O, Jin R, Merikangas KR, Walters EE. Lifetime prevalence and age-of-onset distributions of DSM-IV disorders in the national comorbidity survey replication [Internet]. Arch Gen Psychiatry. Arch Gen Psychiatry; 2005 [cited 2020 Nov 20]. p. 593–602. PMID:15939837

16. Grant BF, Goldstein RB, Saha TD, Patricia Chou S, Jung J, Zhang H, Pickering RP, June Ruan W, Smith SM, Huang B, Hasin DS. Epidemiology of DSM-5 alcohol use disorder results from the national epidemiologic survey on alcohol and related conditions III. JAMA Psychiatry [Internet] American Medical Association; 2015 Aug 1 [cited 2020 Nov 20];72(8):757–766. PMID:26039070

17. Abou-Saleh MT, Janca A. The epidemiology of substance misuse and comorbid psychiatric disorders. Acta Neuropsychiatr England; 2004 Feb;16(1):3–8. PMID:26983871

18. Koenen KC, Ratanatharathorn A, Ng L, McLaughlin KA, Bromet EJ, Stein DJ, Karam EG, Meron Ruscio A, Benjet C, Scott K, Atwoli L, Petukhova M, Lim CCW, Aguilar-Gaxiola S, Al-Hamzawi A, Alonso J, Bunting B, Ciutan M, De Girolamo G, Degenhardt L, Gureje O, Haro JM, Huang Y, Kawakami N, Lee S, Navarro-Mateu F, Pennell BE, Piazza M, Sampson N, Ten Have M, Torres Y, Viana MC, Williams D, Xavier M, Kessler RC. Posttraumatic stress disorder in the World Mental Health Surveys [Internet]. Psychol Med. Cambridge University Press; 2017 [cited 2020 Nov 17]. p. 2260–2274. PMID:28385165

19. Garfin DR, Thompson RR, Holman EA. Acute stress and subsequent health outcomes: A systematic review. J Psychosom Res England; 2018 Sep;112:107–113. PMID:30097129

20. Maercker A, Forstmeier S, Pielmaier L, Spangenberg L, Brähler E, Glaesmer H. Adjustment disorders: prevalence in a representative nationwide survey in Germany. Soc Psychiatry Psychiatr Epidemiol Germany; 2012 Nov;47(11):1745–1752. PMID:22407021
